# A case report of granulomatous amoebic encephalitis by Group 1 *Acanthamoeba* genotype T18 diagnosed by the combination of morphological examination and genetic analysis

**DOI:** 10.1186/s13000-018-0706-z

**Published:** 2018-05-10

**Authors:** Takahiro Matsui, Tetsuo Maeda, Shinsuke Kusakabe, Hideyuki Arita, Kenji Yagita, Eiichi Morii, Yuzuru Kanakura

**Affiliations:** 1 Department of Hematology and Oncology, Osaka University Graduate School of Medicine, 2-2 Yamada-Oka, Suita, Osaka 565-0871 Japan; 2 Department of Pathology, Osaka University Graduate School of Medicine, 2-2 Yamada-Oka, Suita, Osaka 565-0871 Japan; 3 Department of Neurosurgery, Osaka University Graduate School of Medicine, 2-2 Yamada-Oka, Suita, Osaka 565-0871 Japan; 4 Department of Parasitology, National Institute of Infectious Diseases, 1-23-1 Toyama, Shinjuku-ku, Tokyo, 162-8640 Japan

**Keywords:** Granulomatous amoebic encephalitis, Acanthamoeba, Genotype T18, Cerebrospinal fluid

## Abstract

**Background:**

The diagnosis of granulomatous amoebic encephalitis is challenging for clinicians because it is a rare and lethal disease. Previous reports have indicated that *Acanthamoeba* with some specific genotypes tend to cause the majority of human infections. We report a case of granulomatous amoebic encephalitis caused by *Acanthamoeba* spp. with genotype T18 in an immunodeficient patient in Japan after allogenic bone marrow transplantation, along with the morphological characteristics and genetic analysis.

**Case presentation:**

A 52-year old man, who had undergone allogenic bone marrow transplantation, suffered from rapid-growing brain masses in addition to pneumonia and died within 1 month from the onset of the symptoms including fever, headache and disorientation. Infection with *Acanthamoeba* in the brain and lung was confirmed by histological evaluation; immunohistochemical staining and polymerase chain reaction analysis using autopsy samples also indicated the growth of *Acanthamoeba* in the brain. Gene sequence analysis indicated that this is the second documented case of infection with *Acanthamoeba* spp. with genotype T18 in a human host. Postmortem retrospective evaluation of cerebrospinal fluid sample in our case, as well as literature review, indicated that some cases of granulomatous amoebic encephalitis caused by *Acanthamoeba* may be diagnosable by cerebrospinal fluid examination.

**Conclusion:**

This case indicates that *Acanthamoeba* spp. with genotype T18 can also be an important opportunistic pathogen. For pathologists as well as physicians, increased awareness of granulomatous amoebic encephalitis is important for improving the poor prognosis along with the attempt to early diagnosis with cerebrospinal fluid.

## Background

Allogenic hematopoietic stem cell transplantation recipients often develop several opportunistic infections associated with fatal outcomes. Pathogens that cause opportunistic infections include not only bacteria and viruses but also fungi and parasites that do not cause infection in healthy individuals. *Acanthamoeba* is a free-living amoeba that is pathogenic to humans and important as the etiological agent of amoebic keratitis that occurs mainly among contact lens users. In rare cases, however, this pathogen causes an intracranial infection called granulomatous amoebic encephalitis (GAE) mainly in immunocompromised patients. Although early diagnosis is important, the diagnosis of GAE is challenging for clinicians because it is a rare and lethal disease. Moreover, previous reports have indicated that *Acanthamoeba* spp. with some specific genotypes, such as those belonging to morphological Group 2, tend to cause the majority of human infections. We herein describe a fatal case of GAE caused by *Acanthamoeba* spp. with genotype T18 after allogenic bone marrow transplantation with review of the literature.

## Case presentation

**Fig. 1:**
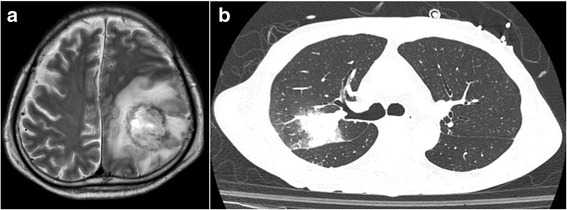
Image inspection findings. **a** T2-weighted magnetic resonance imaging scan of head. **b** Computed tomography scan of lung

A Japanese man who had been diagnosed with aplastic anemia underwent allogenic bone marrow transplantation from an unrelated donor at 51 years of age. After transplantation, he suffered acute graft-versus-host disease with a systemic rash and severe diarrhea. After treatment with adrenocortical steroid and anti-human thymocyte globulin, his symptoms gradually improved, and he was discharged 15 months after transplantation on oral prednisolone 25 mg/day. About 10 days after discharge, he developed fever, headache, and disorientation and was admitted for emergency care in our hospital. Image inspections of the head revealed mass lesions in the left parietal and occipital lobes (Fig. 1a). Diffuse haziness in the upper lobe of the right lung was also observed (Fig. 1b). Our differential diagnoses included cryptococcal meningoencephalitis, bacterial brain abscess, aspergillosis, nocardiosis, tuberculosis, and central nervous system post-transplant lymphoproliferative disorder. Sputum, blood and cerebrospinal fluid (CSF) cultures all yielded negative results. The CSF sample did not show any pathological findings, such as increased number of inflammatory cells or decreased glucose levels (91 mg/dL). Cryptococcal antigen was neither detected in the serum nor CSF. No elevation of plasma beta-D-glucan was observed (2·5 pg/mL). Bronchoscopy, bronchoalveolar lavage, and transbronchial lung biopsy were performed, but none showed specific findings. Although antimicrobial and antifungal agents were administered, his condition worsened, and convulsions with apnea episodes occurred frequently. An emergency craniotomy was performed for the purpose of biopsy and cerebral decompression. Rapidly frozen samples showed necrotic tissues with focal lymphocyte aggregation, which were not helpful for confirming a diagnosis. Three days after the craniotomy, however, hypotension, bradycardia, and pupillary dilatation suddenly occurred without any evidence of intracranial hemorrhage. A brainstem infarction was highly suspected. The patient died after being brain-dead for about 10 days, 4 weeks after the onset of the neurological disorder.

## Pathological findings

**Fig. 2:**
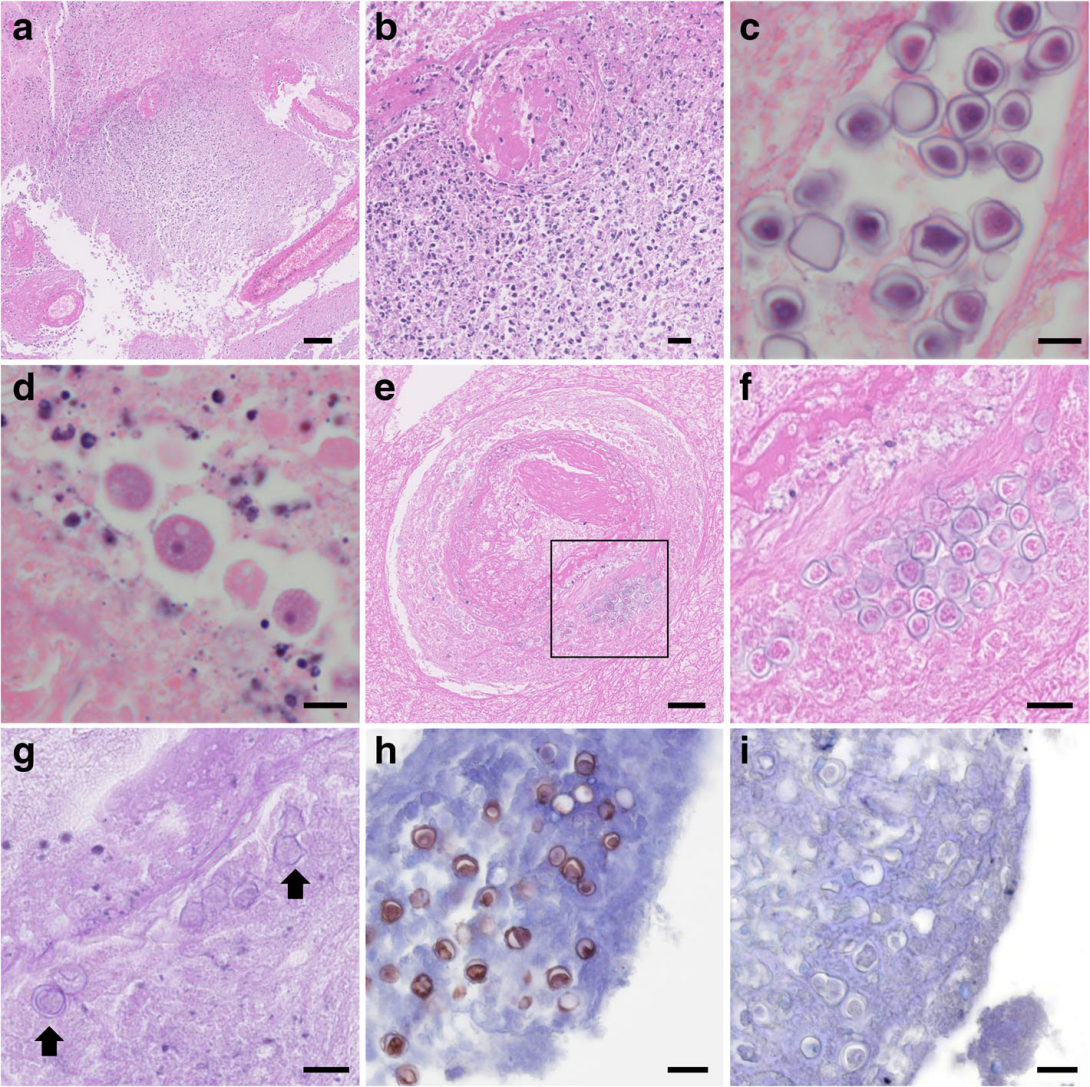
Histopathological findings of brain biopsy sample. **a** and **b** Hematoxylin and eosin staining with low (**a**) and medium magnification (**b**) of the brain biopsy sample. Necrotic tissue with inflammatory cell infiltration and vessel hyalinization was observed. Scale bar: 100 μm (for **a**) and 20 μm (for **b**). **c**, **d**, **e**, **f** and **g** Hematoxylin and eosin staining of amoebic cysts (**c**) and trophozoites (**d**) in the brain biopsy sample. These pathogens were observed in necrotic tissues and cysts were also observed around blood vessels (**e**). **f** shows the magnified image of square area in (**e**). These cysts showed faint positive in Periodic acid-Schiff stain (**g**, arrows). Scale bar: 10 μm (for **c** and **d**), 50 μm (for **e**) and 20 μm (for **f** and **g**). **h** and **i** Immunohistochemical staining of amoebic cysts. The amoebic cysts were positive for antiserum against *Acanthamoeba* (**h**), but negative for *Balamuthia* (**i**). Scale bar: 20 μm

**Fig. 3:**
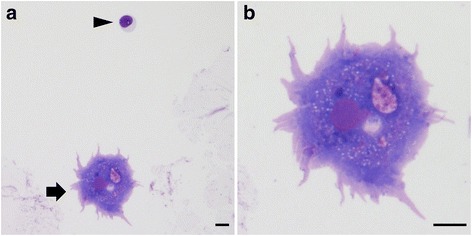
Cytological findings of Group 1 *Acanthamoeba* in CSF. **a** and **b** Giemsa staining of a trophozoite of Group 1 *Acanthamoeba* in the cerebrospinal fluid. Arrow in (**a**) shows a trophozoite and arrow head indicates a lymphocyte. **b** shows the magnified image of (**a**). Scale bar: 10 μm

**Fig. 4:**
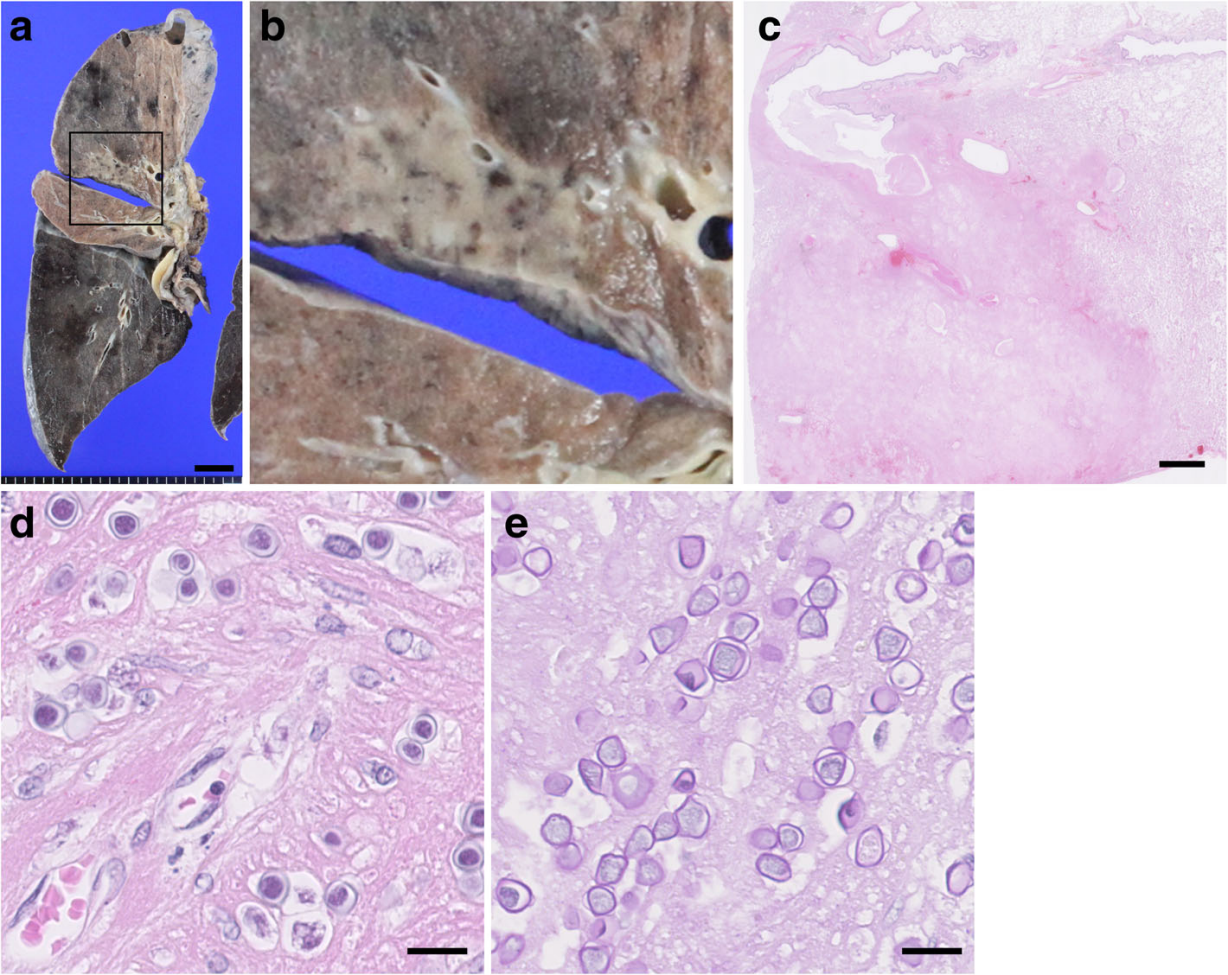
Histopathological findings of right lung. **a** and **b** Gross appearance of coronal section of the right lung. Grayish lesion with clear boundary was observed in the upper lobe of the right lung. **b** shows the magnified image of square area in (**a**). Scale bar: 2 cm. **c** Very low-power field of the right lung. Necrotic lesion with clear boundary was observed. Scale bar: 2 mm. **d** and **e** High-power field of necrotic lesion in the right lung. Amoebic cysts were observed in a part of nectoric lesion (**d**). These cysts showed faint positive in Periodic acid-Schiff stain (**e**). Scale bar: 20 μm

**Fig. 5:**
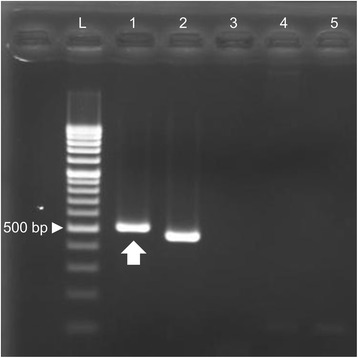
PCR analysis of DNA extracted from brain autopsy sample. Agarose gel electrophoresis of PCR products. L denotes 100-bp ladder and arrowhead indicates 500 base pairs. DNA isolated from the autopsy brain sample (Lane 1, arrow), positive control of *Acanthamoeba* (Lane 2), that of *Balamuthia* (Lane 3), human genome DNA (Lane 4) and negative control (Lane 5) were respectively amplified using the JDP1 and JDP2 primers

Antemortem brain biopsy samples obtained via craniotomy showed diffuse necrotic tissues with inflammatory cell infiltration and vessel hyalinization (Fig. 2a and b), and amoebic cysts and trophozoites were observed in the necrotic tissues (Fig. 2c and d). Amoebic cysts were also observed around the blood vessels (Fig. 2e and f). These cysts showed faint positive after Periodic acid-Schiff staining (Fig. 2g). Immunohistochemical staining of the tissue revealed that the amoebic cysts were positive for antiserum against *Acanthamoeba* (Fig. 2h) but negative for *Balamuthia* (Fig. 2i). A retrospective evaluation of the antemortem CSF sample also revealed only a few trophozoites with Giemsa staining (Fig. 3a and b). The trophozoites measured about 35 μm, exhibiting large blunt-end protrusions, and were morphologically compatible with acanthapodia, particularly those in Group 1 *Acanthamoeba*. From these results, the patient was diagnosed with GAE caused by Group 1 *Acanthamoeba*. General autopsy findings showed that the central nervous tissue was diffusely liquefied with necrosis, and an apparent mass lesion, identified in the initial imaging diagnosis, was not observed. Histologically, most of this liquefied brain tissue consisted of necrotic tissue without significant infiltration of inflammatory cells. From the presence of systemic multiple embolism as well as typical clinical course, we concluded that the brain liquefaction was due to infarction caused by non-bacterial thrombotic endocarditis in the aortic valve. However, amoebic cysts were detected in the necrotic tissue. Moreover, amoebic cysts were also observed in the upper lobe of the right lung, where a grayish lesion with clear boundaries was detected macroscopically (Fig. 4a, b, c, d and e). No obvious amoebae were observed, other than those in the brain and lung. For molecular identification, polymerase chain reaction (PCR) analysis using DNA extracted from the necrotic lesion in the brain tissue was performed. Established PCR primers JDP1 and JDP2, that amplify the 18S ribosomal RNA gene of the genus *Acanthamoeba* [1], successfully amplified the *Acanthamoeba*-specific fragment (Fig. 5). We also amplified and sequenced approximately 2500 base pairs of 18S ribosomal RNA gene fragments. A BLAST (Basic Local Alignment Search Tool) analysis of the partial 18S ribosomal RNA gene sequence showed the highest homology (98%) with *Acanthamoeba* sp. CDC: V621 strain, type T18, which morphologically belongs to Group 1 [2].

## Discussion

The *Acanthamoeba* spp. are free-living amoebae that are pathogenic to humans and ubiquitous in natural environments. They trigger either keratitis, primarily among contact lens users, or cerebral lesions as opportunistic infections primarily in immunocompromised patients, called GAE. In cases of GAE, *Acanthamoeba* causes an infection through either ulcerated skin or the lower respiratory tract and disseminate hematogenously to the central nervous system. Almost all cases of GAE have been diagnosed by postmortem examination, due to its low incidence and fulminant clinical course. The members of the genus *Acanthamoeba* are classified into three groups (Groups 1–3) based on morphological characteristics [3], and in molecular identification, they are divided into 20 sequence types (T1–T20) based on the 18S ribosomal RNA gene sequence diversity [4]. The sequence types generally correlate well with the morphologically derived species. Importantly, the vast majority of *Acanthamoeba* associated with human infections belong to Group 2, primarily with sequence type T4 [5]. In contrast, *Acanthamoeba* with Group 1 morphology, such as in this case, have been isolated primarily from the environment and are considered to have extremely low pathogenicity in humans. As far as we know, there has only been one case of GAE caused by *Acanthamoeba* with Group 1 morphology [2]. The presented case indicates that immunocompromised hosts, such as transplant patients on medication, should be carefully monitored for several opportunistic infections including GAE and that pathogens with extremely low human pathogenicity, such as Group 1 *Acanthamoeba*, may cause fulminant infections in severely immunodeficient individuals.

In this case, extensive liquefaction of the brain was observed at autopsy. However, there have been no reports of cases with liquefied brain tissue caused by GAE, and foci of hemorrhagic necrosis were observed macroscopically in most autopsy cases [6–9]. Moreover, in this case, there were multiple infarct lesions with thrombus including myocardium and spleen in addition to non-bacterial thrombotic endocarditis in the aortic valve. The clinical course also suggested sudden onset of extensive cerebrovascular disease. For these reasons, we concluded that the extensive liquefaction was caused by cerebral infarction and associated brain death, not by GAE.

Literature review suggests failed treatment of all patients with GAE caused by *Acanthamoeba* in the field of hematopoietic cell transplantation. However, some studies have reported that either skin biopsy or CSF analysis can be used in the antemortem diagnosis of GAE. Kaul et al. reported a GAE case caused by *Acanthamoeba*, diagnosed antemortem by performing an ulcerated skin lesion biopsy, although the patient did not respond to the treatment [8]. When cutaneous ulcerative lesions are found in immunocompromised patients with neurological symptoms, skin biopsy should be performed because *Acanthamoeba* may enter through the skin ulcer and amoebic trophozoites and cysts may be histologically observed in the suppurative tissue. If the disease is localized to the skin and diagnosed immediately, intensive treatment may lead to successful outcome. In fact, Walia et al. reported a case of successful treatment for cutaneous *Acanthamoeba* infection [10]. However, when a skin lesion is not detected and amoebae are suspected to have entered through the respiratory tract, the only way to diagnose the infection is by examining the CSF. In our case, a retrospective microscopic evaluation of the antemortem CSF sample revealed the presence of trophozoites. This case demonstrated that skilled microscopic observation enables the prompt diagnosis of GAE and that a molecular analysis provides definite evidence of amoebic infection. Even if amoebae are not evident in CSF samples, CSF may contain amoeba DNA from extensive necrosis of brain tissue or lysed amoeba cells. Although PCR analysis using CSF specimen was not performed in our case, Yagi et al. suggested that *Acanthamoeba* DNA can be detected in CSF samples by performing PCR analysis [11]. A sufficient amount of the CSF sample and careful analysis, such as using PCR, may be the only way to diagnose GAE in the early stage. CSF culture for *Acanthamoeba* requires specific culture conditions and is only applicable in limited laboratories [12].

The prognosis of GAE is very poor, and no definitive treatment protocol has been established yet. A previous report indicated that only a few drugs have demonstrated in vitro activity against *Acanthamoeba* and have resulted in the successful treatment of a few patients: azoles, rifampicin, pentamidine, sulfadiazine, azithromycin, caspofungin, etc. [13]. The clinical usefulness of subclassification of *Acanthamoeba* also remains unknown at this time. However, as the utility of molecular biological analysis have increased in clinical medicine, it is estimated that the subdivision of *Acanthamoeba* will become increasingly useful in the future for both epidemiological analysis and development of effective therapies. This case indicates that morphological classification by examination of CSF samples is useful, as well as genome sequence classification. Accumulation of clinical data along with the attempt to diagnose GAE in detail is indispensable for the establishment of therapeutic strategies and the improvement of the poor prognosis of GAE.

## Conclusion

This case suggests that *Acanthamoeba* spp. with genotype T18 can also be an important opportunistic pathogen in infections such as GAE in humans. The number of *Acanthamoeba* infections has gradually increased worldwide [3] and opportunistic infections have also increased due to HIV/AIDS and advances in modern medicine, such as chemotherapy and transplantation. The decline of autopsy may cover the undiagnosed cases. GAE caused by *Acanthamoeba* should be considered when assessing an immunocompromised host with neurological abnormalities. For pathologists as well as physicians, increased awareness of this rare but lethal disease is important for improving the poor prognosis along with the attempt to early diagnosis with CSF.

## Abbreviations

CSF: Cerebrospinal fluid
GAE: Granulomatous amoebic encephalitis
PCR: Polymerase chain reaction
